# Essential properties and pitfalls of colorimetric Reverse Transcription Loop-mediated Isothermal Amplification as a point-of-care test for SARS-CoV-2 diagnosis

**DOI:** 10.1186/s10020-021-00289-0

**Published:** 2021-03-26

**Authors:** Bruna de Oliveira Coelho, Heloisa Bruna Soligo Sanchuki, Dalila Luciola Zanette, Jeanine Marie Nardin, Hugo Manuel Paz Morales, Bruna Fornazari, Mateus Nóbrega Aoki, Lucas Blanes

**Affiliations:** 1 Laboratory for Applied Science and Technology in Health, Carlos Chagas Institute, Oswaldo Cruz Foundation (Fiocruz), Prof Algacyr Munhoz Mader 3775 Street, Curitiba, Paraná 81350-010 Brazil; 2 Erasto Gaertner Hospital, Dr. Ovande do Amaral 201 Street, Curitiba, Paraná 81520-060 Brazil

**Keywords:** RT-LAMP, SARS-CoV-2, Point of Care

## Abstract

**Background:**

SARS-CoV-2 *Reverse Transcription Loop-mediated Isothermal Amplification* (RT-LAMP) colorimetric detection is a sensitive and specific point-of-care molecular biology technique used to detect the virus in only 30 min. In this manuscript we have described a few nuances of the technique still not properly described in the literature: the presence of three colors clusters; the correlation of the viral load with the color change; and the importance of using an internal control to avoid false-negative results.

**Methods:**

To achieve these findings, we performed colorimetric RT-LAMP assays of 466 SARS-CoV-2 RT-qPCR validated clinical samples, with color quantification measured at 434 nm and 560 nm.

**Results:**

First we determinate a sensitivity of 93.8% and specificity of 90.4%. In addition to the pink (negative) and yellow (positive) produced colors, we report for the first time the presence of an orange color cluster that may lead to wrong diagnosis. We also demonstrated using RT-qPCR and RT-LAMP that low viral loads are related to Ct values > 30, resulting in orange colors. We also demonstrated that the diagnosis of COVID-19 by colorimetric RT-LAMP is efficient until the fifth symptoms day when the viral load is still relatively high.

**Conclusion:**

This study reports properties and indications for colorimetric RT-LAMP as point-of-care for SARS-CoV-2 diagnostic, reducing false results, interpretations and optimizing molecular diagnostics tests application.

**Supplementary Information:**

The online version contains supplementary material available at 10.1186/s10020-021-00289-0.

## Background

The emergence of COVID-19 pandemic caused by SARS-CoV-2 began in Wuhan, China in December 2019, and since then this virus has infected over 89 million people around the globe. Despite its low mortality rate, according to the World Health Organization (WHO) its rapid spread has caused the death of more than a 1.9 million people in 2020 (https://covid19.who.int/, last accessed January 12, 2021). SARS-CoV-2 is a positive, single-stranded RNA virus related to the group of severe acute respiratory syndrome viruses. COVID-19 may develop from typical flu symptoms to severe pneumonia in 2 days to 2 weeks after first contact with the virus (Zhou et al. 2020). People with underlying medical conditions and the elderly are especially at risk to develop the most severe form of the disease (Emami et al. 2020). Currently, there is no specific treatment for COVID-19. Meanwhile, it is necessary to control the virus spread with a strong and rapid health vigilance and a reliable diagnostic tool for large-scale screening (Ali et al. 2020). At the moment, Real time reverse transcription-PCR (RT-qPCR) is the gold standard method for COVID-19 diagnosis. This molecular biology technique is able to detect the virus in approximately 2 h with high sensitivity and specificity. RT-qPCR simultaneously detects viral and human RNA, using specific primers for the viral RNA and for a housekeeping human gene (Corman et al. 2019; Lübke et al. 2020). Despite its high efficiency, this method has some limitations for large-scale testing, such as high costs, requirement of special facilities and trained personnel.

The implementation of a point-of-care approach is of urgent importance to control the pandemics, especially in less developed countries where there are limited resources. An efficient point-of-care test must be rapid, accessible, sensitive, specific and able to be performed without the necessity of special facilities or equipment (Gouilh et al. 2020; Osterdahl et al. 2020). The priority would be to apply this approach in airports, hospitals and in rural areas (Mautner et al. 2020).

The LAMP method was developed during 2000 by Notomi et al., where the nucleic acid amplification is carried by DNA polymerase from *Bacillus stearothermophilus* (Bst), an enzyme with both DNA polymerase and reverse transcriptase activity. This method is performed with 4 primers, two inner (FIP and BIP) and two outer (F3 and B3) primers, or usually 6 primers, the same 4 and more two loop primers (LF and LB), to improve and accelerate amplification. The DNA amplification in LAMP method is initiated by multiple primers simultaneously, making this an efficient and sensible technique. Detection method involves such agarose gel electrophoresis, colorimetric naked eye, sample turbidity and fluorescence. The reverse-transcription loop-mediated isothermal amplification (RT-LAMP) has been extensively applied for the diagnosis of many pathogens (Ahn et al. 2019; Imai et al. 2006; Sukphattanaudomchoke et al. 2020; Techathuvanan and D’Souza 2020), and can amplify RNA molecules in usually 30 min with high sensitivity and specificity in a single temperature, ranging from 60 to 65 °C (Notomi et al. 2000; Tomita et al. 2008; Ushikubo 2004). Due to this characteristic the test can be performed in heat blockers or any other device able to maintain a single temperature for the time needed. It is a cheap and reliable assay, that does not require special personnel and facilities. Therefore, RT-LAMP has all the necessary requirements for point-of-care applications, even more than reports demonstrating the use of RT-LAMP with crude clinical samples such as saliva, or with minimal sample processing (Augustine et al. 2020; Yamazaki et al. 2021; Howson et al. 2021; Chow et al. 2020; Wei et al. 2021). Moreover, immunological lateral-flow as point-of-care test for SARS-CoV-2 usually present low accuracy for IgM detection (Chembio Diagnostic system DPP^®^ 2020; Haguet et al. 2021, Dortet et al. 2021). Again, lateral-flow as point-of-care targeting SARS-CoV-2 antigen relies low sensitivity and accuracy (Wise 2020; Coris BioConcept 2020; JMH Labour and Welfare 2020). More than that, lateral-flow tests allow just one sample per test while RT-LAMP multiple samples can be performed in a single run.

RT-LAMP for SARS-CoV-2 detection has already been widely studied and reported in literature (Gouilh et al. 2020; Lalli et al. 2020; Thi et al. 2020). When the primers are well designed, the test is highly specific and does not show cross reactivity with other pathogens (Meagher et al. 2018). RT-LAMP is also able to detect low copies of the virus, demonstrating its extremely promising sensitivity (Huang et al. 2020; Yan et al. 2020). Many authors optimize the reactions using pH indicators and fluorescent dyes to visualize the result by naked eye, eliminating the agarose gel step (Mautner et al. 2020; Zhang et al. 2020). This approach turns RT-LAMP cheaper and even more accessible. With that in mind, RT-LAMP is one of the most relevant point-of-care tools reported in literature recently. However, many authors do not associate the reaction with the use of an internal control as in RT-qPCR, which in our opinion compromises the fidelity of the results.

The internal control is used to prove the presence of RNA in the sample. Usually, it is used in a duplex, where human and viral RNA are detected simultaneously. The use of internal controls is mainly used in RT-qPCR but not in other assays (Vandesompele et al. 2002). If a sample turns positive for SARS-CoV-2 but not for the internal control, it is considered an invalid result. It indicates a problem either in the RNA extraction process or in sample collection. The use of an internal control is of paramount importance to minimize the occurrence of technical errors and false results. Generally, authors who suggest RT-LAMP as a point-of-care approach do not use internal controls, which seriously compromises the quality of the results obtained.

In this work, we propose a COVID-19 diagnosis assay for fast detection of SARS-CoV-2 virus based on RT-LAMP with the addition of an internal control. In both cases, the whole reaction can be performed in only 30 min, and positive or negative samples could be easily detected by naked eye through simple color change. We demonstrated how the internal control must be applied for results validation. These assays were validated using 466 clinical samples with 93.8% sensitivity. We further evaluated Ct cut-off values to improve RT-LAMP performance, its relation with high and low viremia, and how Ct values are determinant to establish a limitation for the colorimetric test.

## Methods

### Samples and ethical statement

Four hundred sixty-six clinical samples were collected from SARS-CoV-2 symptomatic patients from May to November 2020 in Erasto Gaertner Hospital (Curitiba—Brazil) after Local Ethics Committee approval (CAAE 31592620.4.3001.5248 and 31592620.4.1001.0098). All samples collection and experimental conduction were carried out in accordance with relevant guidelines and brazilian regulations. All recruted patients have written a consent. On the first day in the hospital, two nasal and one oral rayon-swabs were collected in 3 mL of 1 × PBS. The RNA extraction was performed with QIAmp Viral RNA Mini Kit (Qiagen) as described by the manufacturer. Symptom days were collected as patient self-declaratory data.

### Real-time PCR

RT-qPCR protocol (Corman et al. 2020) was used as a gold-standard method for SARS-CoV-2 detection. Primers and probes for SARS-CoV-2 E-gene (FAM) and human RNase P (HEX) were acquired from Integrated DNA Technoogies (IDT, United States), and resuspended in nuclease-free water (Invitrogen, United States). Reactions were performed with SuperScript™ III Platinum™ One-Step qRT-PCR Kit (ThermoFisher, United States) and 5 μL of RNA at 50 °C for 30 min, 95 °C for 5 min, 45 cycles of 95 °C for 15 s and 58 °C for 30 s using the LightCycler96 platform (Roche, Germany). As positive control, we used RNA extracted from supernatants of SARS-CoV-2 cultured in Vero cells using QIAamp^®^ RNA viral Mini Kit (Qiagen, Germany), following the manufacturer’s instructions. The RNA and consequent viral genome quantification were performed by real-time PCR on LightCycler^®^ 96 (Roche, Germany) using the same primers and probes and an E-gene standard curve (SARS-CoV Frankfurt1; Full virus RNA, Lot2; Institute of Virology, Charité). Samples with a Ct lower than 35 in E-gene were considered SARS-CoV-2 positives. To validate the procedure, the samples had to amplify the internal control RNase P at a maximum Ct of 35.

### RT-LAMP primers, assays and quantification

After literature screening for SARS-CoV-2 colorimetric RT-LAMP primer sets we selected those described by Rabe and Cepko 2020, named Orf1a-HMS, with FIP and BIP primers with 4 thymidine residues inserted in the middle (Orf1a-HMSe) (see Additional file 1: Table S1). For internal control, the primer set was designed by PrimerExplorer V5 (https://primerexplorer.jp/e/) for human 18S RNA sequence. Reactions were performed with WarmStart^®^ Colorimetric LAMP 2X Master Mix (NEB, England), in a volume of 25 μL with 6 μL of primer mix (FIP and BIP at 1.6 μM each, FOP and BOP at 0.2 μM each and FL and BL at 0.4 μM each) and 5 μL of RNA sample. The reactions were incubated at 65 °C for 30 min using the ProFlex PCR System (Applied Biosystems, United States) and immediately after finishing the reaction the vials were incubated on ice. For clinical samples performed for both SARS-CoV-2 and internal control, two separate tubes were used for colorimetric RT-LAMP. As positive control, samples of 10^5^ SARS-CoV-2 copies were used as described in the previous section. As non-template control (NTC) 5 μL of nuclease-free water was used. All reactions were submitted to 2% agarose gel electrophoresis (100 V) for 45 min, stained with ethidium bromide and visualized using an UV transilluminator (L-Pix Chemi, Loccus, Brazil). For color quantification, 20 μL of the reaction were pipetted in a 384-well plate and the optical density was measured at 434 nm and 560 nm using a BioteK Synergy reader. To obtain the *Δ*OD (color change difference), absorbance at 560 nm was subtracted from the one at 434 nm.

### Statistical analysis

All graphics and data obtained were analyzed using GraphPad Prism software version 7.0 (GRAPH PAD software Inc, California, USA). Specificity of the RT-LAMP assay was calculated as the fraction of RT-qPCR negative samples that were also negative in the RT-LAMP assay. The sensitivity for Ct interval (Ct < 30 or Ct < 35) was calculated as the fraction of all samples that showed an RT-qPCR Ct value in that range that was also positive in the RT-LAMP assay. Statistical t-test was calculated as unpaired and parametric test, with a statistically significant difference of p < 0.05. Receiver Operating Characteristic (ROC) curve was calculated by grouping the *Δ*DO of SARS-CoV-2 colorimetric RT-LAMP of RT-qPCR positive and negative SARS-CoV-2 samples with 95% of confidence interval and a statistically significant difference of p < 0.05. Correlation between symptoms days and RT-qPCR SARS-CoV-2 detection and colorimetric RT-LAMP results were calculated using unpaired parametric t-test and a statistically significant difference of p < 0.05.

## Results

### SARS-CoV-2 detection by RT-qPCR

From the 466 clinical samples, 250 were negative and 216 were positive for SARS-CoV-2 according to RT-qPCR, with an E-gene Ct ranging from 13.38 to 34.65. For SARS-CoV-2 positive samples, 74.1% presented Ct lower than 30, while 25.9% had Ct values between 30 and 35. All 466 samples were positive for RNAse P internal control, with Ct ranging from 14.24 to 34.91 (see Additional file 2: Fig. S1).

### Validation of RT-LAMP for SARS-CoV-2 detection

**Fig. 1:**
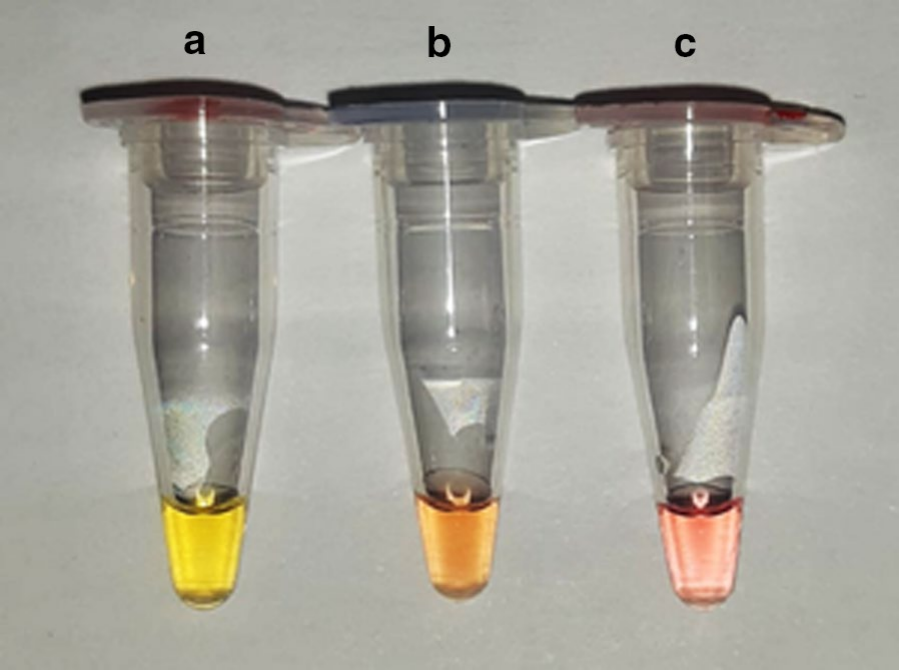
Colorimetric RT-LAMP color display. Tube (**a**) positive (**b**) intermediate and (**c**) negative. This analysis was made by naked eye as in a real case scenario

After obtaining the RT-qPCR results, SARS-CoV-2 colorimetric RT-LAMP was performed with the same 466 clinical samples, aiming to determine RT-LAMP diagnostic sensitivity and specificity. Several RT-LAMP primer sets have been described for SARS-Cov-2 detection in literature with promising sensitivity and specificity. After a screening and previous evaluations from our group, it was decided to proceed with the primers designed by Rabe and Cepko (Rabe and Cepko 2020). A visible color change from pink to yellow is observed when the amplification of the target sequence has taken place, due to the presence of phenol red, a pH indicator presented in the reaction. When the reaction occurs, the pH drops from ~ 8 (pink) to ~ 6 (yellow) changing the color. During the testing process we verified that many samples presented an intermediate orange color that could not be easily distinguished. As a naked eye interpreted test, we clustered the samples in positive, intermediate and negative, according to the comparison with positive and non-template controls (Fig. 1). Most of the reactions with an intermediate orange color corresponded to positive samples with low viremia, as detected by RT-qPCR and demonstrated in the next sections. It is important to notice that negative samples may display the same intermediate color as those low-viremia samples. Under our knowledge, no previous reports described the existence of a third color cluster as a weakness of colorimetric RT-LAMP method for SARS-CoV-2.

**Fig. 2:**
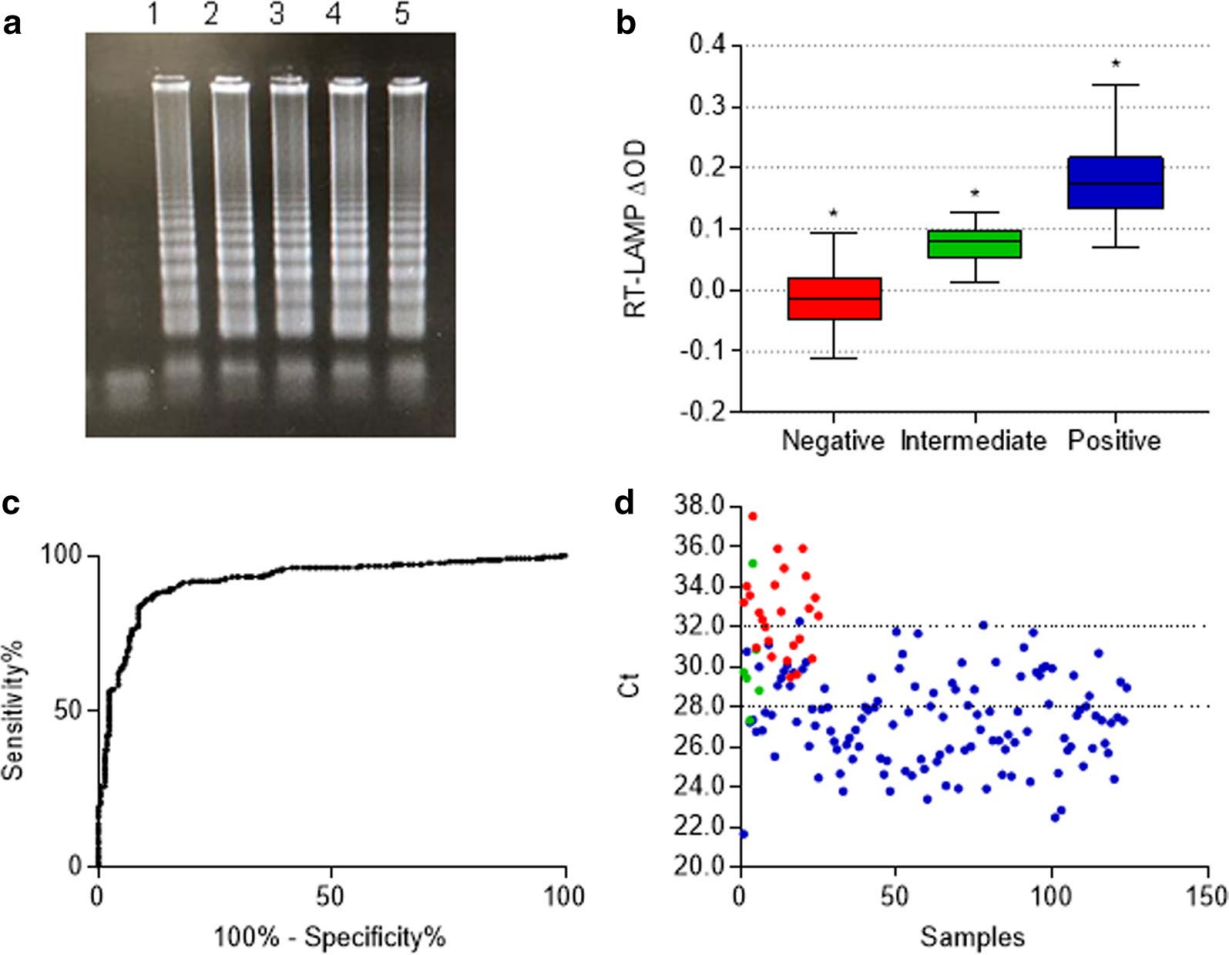
RT-LAMP validation results. **a** Electrophoresis of RT-LAMP products. RT-LAMP reaction products were analyzed on a 2% agarose gel and the DNA was stained with ethidium bromide. The typical band pattern of a RT-LAMP reaction was visible in positive samples that showed a color change from pink to yellow. Lane 1 = NTC and lanes 2–6 = positive samples. **b** Colorimetric readout after 30 min of RT-LAMP reaction. After the reaction, the optical densities were measured at 434 nm and 560 nm using a BioteK Synergy reader. To obtain the *Δ*OD (color change difference), the absorbance at 560 nm was subtracted from the one at 434 nm. The line inside the box indicates the median and the whiskers extend either to the minimum and maximum values. * Indicates difference (p > 0.001) between groups (unpaired, t-test). **c** Receiver Operating Characteristic (ROC) curve for SARS-CoV-2 colorimetric RT-LAMP with area under the curve of 0.92 (*p* < 0.0001) and 95% confidence interval (CI) 0.8924 to 0.9483. **d** Detection of SARS-CoV-2 through colorimetric RT-LAMP assay and comparison between colorimetric RT-LAMP results and RT-qPCR Ct values. Between the dotted lines is a conflicted area where most of the intermediate samples are found

As demonstrated in Table 1, the overall diagnostic sensitivity and specificity of colorimetric RT-LAMP when compared to RT-qPCR was 76.9% and 90.4%, respectively. It is important to emphasize that colorimetric RT-LAMP samples were considered positive when they presented a clear yellow color similar to positive control. All these samples were also positive after 2% agarose gel electrophoresis stained with ethidium bromide (Fig. 2a).

**Table 1:** Summary of RT-LAMP results testing 466 clinical samples

	Positive	Negative
	Ct < 35	Ct > 35
RTq- PCR		
Positive	76.9%	5.2%
	n = 166	n = 13
Negative	10.6%	90.4%
	n = 23	n = 226
RT-LAMP		
Intermediate	12.5%	4.4%
	n = 27	n = 11
Total	46.4%	53.6%
	n = 216	n = 250

After evaluating the sensitivity and specificity rates obtained by colorimetric RT-LAMP in the literature, we decided to stratify the samples in two groups, high (Ct < 30) and low (Ct > 30) viremia, in order to better evaluate the assay performance, method properties and its limitations (Fowler et al. 2020; Thi et al. 2020). After this adjustment, the assertiveness of the test increased considerably, reaching a sensitivity of 93.8% as shown in Table 2. Although the sensitivity was 93.8%, only 0.6% of samples were false negatives, since 5.6% of the results were classified as intermediate. Regarding the specificity, the number of false positives was 5.2% and the percentage of negative samples that were detected as intermediate was 4.4%.

**Table 2:** RT-LAMP results testing 466 clinical samples stratified by Ct values

	Positive	Positive	Negative
		Low viral load	
	Ct < 30	Ct 30–35	Ct > 35
RTq-PCR			
Positive	93.8%	28.6%	5.2%
	n = 150	n = 16	n = 13
Negative	0.6%	39.3%	90.4%
	n = 1	n = 22	n = 226
RT-LAMP			
Intermediate	5.6%	32.1%	4.4%
	n = 9	n = 18	n = 11
Total	34.3%	12.0%	53.6%
	n = 160	n = 56	n = 250

This cut-off value was decided after observation of unspecific amplifications with Ct > 35 and by previous experiments of our group demonstrating that our RT-qPCR protocol showed 100% of sensitivity for 100 copies of SARS-CoV-2 with a mean Ct close to 35 (data not shown). If we sum positive and intermediate colorimetric RT-LAMP results the sensitivity increases to 89.4% for overall RT-qPCR positive samples (76.9% positive + 12.5% intermediate) and to 99.4% when considered samples with Ct < 30 (93.8% positive + 5.6% intermediate). In a similar way, if we sum negative and intermediate colorimetric RT-LAMP results, the specificity increases to 94.8%. In order to increase the reliability of color determination and interpretation, we performed color quantification by spectrophotometry. The color change rearranges the wavelength peaks at 434 nm and 560 nm from phenol red that drastically changes during the acidification reaction. Although naked eye visualization is sufficient to detect whether a sample is positive or negative in the colorimetric RT-LAMP, we decided to measure the absorbance difference (*Δ*OD) of the 434 nm and 560 nm peaks of phenol red. When amplification occurs, it leads to an acidification of the reaction that changes the peaks of the dye and makes quantification possible. With this approach we observed that the three colors clusters are clearly distinguished and significantly different from each other (Fig. 2b). These results demonstrate that not all colorimetric RT-LAMP samples are able to be easily and uniquely discriminated between positive and negative samples.

To better evaluate SARS-CoV-2 colorimetric RT-LAMP assay here described, we performed a Receiver Operating Characteristic (ROC) curve, illustrating the diagnostic ability and connection between diagnostic sensitivity and specificity. As demonstrated in Fig. 2c, this ROC curve returns an area under the curve (AUC) of 0.92, indicating that this method presents an excellent performance (Fig. 2c).

For an improved comprehension of color determination, viral load and colorimetric RT-LAMP, we demonstrate the correlation between RT-qPCR SARS-CoV-2 positive samples and colorimetric RT-LAMP results (Fig. 2d). We showed and determined a “gray zone” between Ct 28 and 32 in which positive, intermediate and negative colorimetric RT-LAMP samples can be observed. All samples with Ct values lower than 28 were positive in colorimetric RT-LAMP, while Ct values greater than 32 were mostly negative. Thus, we propose that RT-qPCR CT values of 28 or less represent the method safe zone, while higher values may produce inconsistent results.

This safe zone was also demonstrated when the assay repeatability was assessed with 172 samples. From 90 SARS-CoV-2 positive samples, it was found 23 inconsistencies, but all of them had a Ct greater than 28 for the E-gene, while all 50 samples with Ct lower than 28 showed 100% reproducibility. Regarding 82 SARS-CoV-2 negative samples (Ct > 35), 12 showed inconsistent results, with 8 samples varying between negative and intermediate. It was also observed that intermediate samples provided the less consistent results, reinforcing the proposed colorimetric RT-LAMP safe zone. However, we believe that some of the mismatch results may be due to low quantity or quality of the genetic material after RNA extraction and suggest that before performing RT-LAMP reactions it is necessary to ensure that there is good quality and quantity of RNA.

### Internal control

**Fig. 3:**
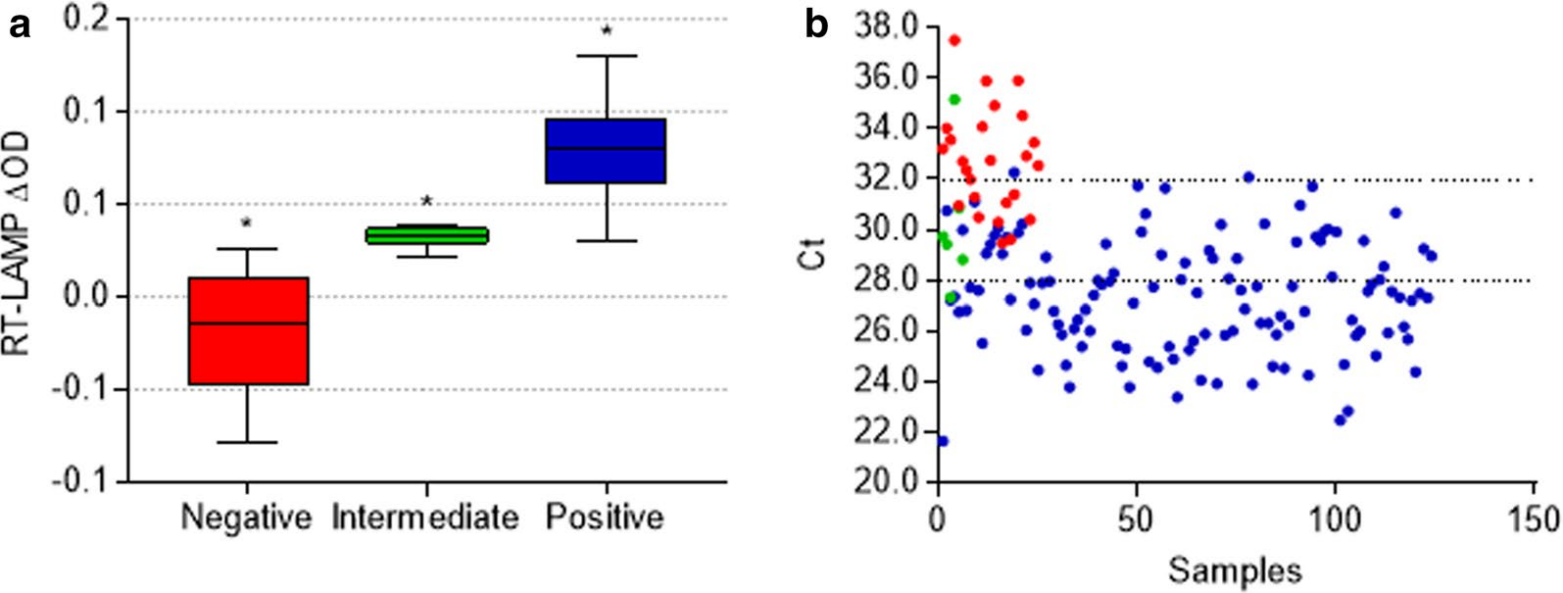
RT-LAMP internal control results. **a** Colorimetric readout after 30 min of 18S internal control RT-LAMP reaction. Samples were pipette in a 384-well plate and the optical density was measured at 434 nm and 560 nm using a BioteK Synergy reader. To obtain the *Δ*OD (color change difference), absorbance at 560 nm was subtracted from the one at 434 nm. The line inside the box indicates the median and the whiskers extend either to the minimum and maximum values. * Indicates difference (p > 0.001) between groups (unpaired, t-test). **b** Detection of internal control 18S by RT-LAMP assay. Comparison between colorimetric RT-LAMP results and RT-qPCR CT values (RNAse P)

To perform RT-LAMP with internal control, the primer set designed for human internal control was within the 18S ribosomal RNA. The internal control reactions for colorimetric RT-LAMP were performed in 155 clinical samples and divided in two groups according to RT-qPCR internal control performance: 115 samples with Ct < 30 and 40 samples with Ct > 30. Sample amplifications were also confirmed by agarose gel electrophoresis stained with ethidium bromide (data not shown). As demonstrated in Table 3, we observed 94.8% sensitivity for reactions with Ct < 30, in which the colorimetric RT-LAMP was able to detect 109 out of 115 samples, with only 2 negative samples and 4 intermediate samples. When the second cluster was analyzed, i.e. 40 samples with Ct > 30, only 15 were positive for internal control in colorimetric RT-LAMP, 23 were negative and 2 intermediate. Again, the reaction color was measured by spectrophotometry, demonstrating a statistical significance between positive, intermediate and negative samples (Fig. 3a).

**Table 3:** RT-LAMP detection of 18S gene used as internal control

	CT RNAse P	
	CT < 30	CT > 30
RTq-PCR		
Positive	94.8%	37.5%
	n = 109	n = 15
Negative	1.7%	57.5%
	n = 2	n = 23
RT-LAMP 18S		
Intermediate	3.5%	5.0%
	n = 4	n = 2
Total	74.2%	25.8%
	n = 115	n = 40

The results of the colorimetric RT-LAMP with internal control reinforce that this method has a higher sensitivity only for samples with Ct < 30. In agreement with data previously shown for colorimetric RT-LAMP for SARS-CoV-2 detection, clinical samples with low RNA amount, represented by Ct > 30 demonstrated a high rate of negative results.

The importance of internal control in colorimetric RT-LAMP is clearly visualized when the result is plotted against RT-qPCR internal control Ct. The great majority of RT-qPCR samples with internal control Ct above 32 returns negative colorimetric RT-LAMP results (83%). Samples with internal control Ct lower than 28 are mostly positive (98.8%), while samples with Ct between 28 and 32 can potentially return all 3 results possibilities (Fig. 3b).

To illustrate the essential role of internal control for colorimetric RT-LAMP as a point-of-care test, Table 4 displays RT-qPCR Ct for E-gene and RNAse P (internal control) for SARS-CoV-2 detection using some of the samples tested in this report. As it was previously demonstrated, samples with Ct > 30 may produce false-negative results in colorimetric RT-LAMP. When all these 12 samples were performed in colorimetric RT-LAMP just for SARS-CoV-2 detection alone without an internal control, all returned a false-negative test. When the internal control was also performed, it returned a negative result, which clearly indicates low RNA amount or quality, that invalidates the test and avoids false-negative results that would be generated if the internal control was not assessed. Similar situations will occur in point-of-care application of colorimetric SARS-CoV-2 RT-LAMP.

**Table 4:** RT-qPCR SARS-CoV-2 positive clinical samples indicating both E-gene and RNAse P (internal control) Cts

Sample	RT-qPCR (Ct)	
	E-gene	Rnase P
26	34.7	31.3
43	34.4	33.2
55	34.5	30.0
86	31.4	30.0
96	34.3	30.8
162	34.4	29.4
201	29.5	29.8
232	33.0	34.9
242	33.3	31.0
276	29.0	29.1
360	29.7	30.9
361	33.4	30.3

### Days of symptoms

The correlation between the number of days after symptoms onset (self-reported) and the results from RT-qPCR and colorimetric RT-LAMP was conducted in positive SARS-CoV-2 patients, to assess a more in-depth analysis of the assay performance. Table 5 and Additional file 3: Fig. S2 shows RT-qPCR results divided in two groups: one with Ct < 30 and the other with Ct between 30 and 35; and the paired data of self-reported number of days after symptoms onset. There was a significant difference in the mean number of days after symptoms onset between the two Ct groups, with a mean of 5.53 and 7.28 days for Ct > 30 and Ct 30–35, respectively. More importantly, similar results were obtained for colorimetric RT-LAMP and self-reported number of days after symptoms onset, which showed a significant difference between positive and intermediated/negative groups, with mean of 5.36 and 7.63 days, respectively.

**Table 5:** Association between self-reported days of symptoms and RT-qPCR SARS-CoV-2 positive patients and colorimetric RT-LAMP performance

Assay type	Group	Samples	Days of symptoms
RT-qPCR	Ct < 30	117	5.53 ± 0.36*
	Between 30 and 35	39	7.28 ± 0.58
Colorimetric RT-LAMP	Positive	115	5.36 ± 0.35**
	Intermediate/negative	41	7.63 ± 0.59

RT-qPCR was divided into Ct < 30 and between 30 and 35

*p < 0.05; **p < 0.01

## Discussion

During the colorimetric RT-LAMP it was possible to indicate some technical variables as potentially responsible for the origin of intermediate color reactions, such as temperature, time and primer dimer formation. When the test temperature is above 65 °C it was observed that even the no template control (NTC) color was affected, but it did not happen when the temperature was below 65ºC. This observation indicates a direct relationship between temperature and primer dimer formation in our study. It also emphasizes that RT-LAMP needs to be performed in a device able to maintain a precise constant temperature. There are works on literature reporting how LAMP primers (FIP and BIP) tend to create dimers due to their long sequences (Gao et al. 2019; Meagher et al. 2018). Therefore, RT-LAMP primers must be extensively tested before their diagnostic use. After the established reaction time of 30 min in this study, the samples were immediately put in ice. This extra step was implemented after the observation that reactions resting for longer than 30 min had increased nonspecific amplifications. Fowler et al. 2020 had the same conclusion and optimized the reaction time for only 16 min. The decrease in temperature was used to stop the enzyme activity and it was perceptible that the reaction colors were enhanced when in contact with ice (data not shown).

Regarding the color quantification, according to Thi et al. 2020 it is useful to determine RT-LAMP exact sensitivity. The numbers support the naked eye analysis, contributing to the test credibility and decreasing its subjectivity. Still, this quantification improvement is not explored by RT-LAMP studies. Our results clearly exhibit the intermediate range, where samples are harder to classify and thus generate indeterminate results. During the experiments, it was noticed that this range changes according to the lot of master mix used.

The Ct cut-off was determined by our group after unspecific amplifications started to occur. Fowler et al. 2020 performed a 33 Ct cut-off and obtained 97% of sensitivity and 99% of specificity in 16 min. The author emphasizes and recommends the importance of detection within this time limit to avoid nucleic acid degradation. Osterdahl et al. 2020 does not report a Ct cut-off for their RT-LAMP reaction, but the authors reported a sensitivity of 80% and a specificity of 73%, a test performance that is considered low when compared to other studies. A suggested detection limit of Ct < 30 for colorimetric RT-LAMP was proposed by Thi et al. 2020 in clinical trials. After the evaluation of 768 samples the authors observed that samples with Ct > 30 remained negative or after 30 min unspecific amplification started to occur. Schermer et al. 2020 established a Ct < 30 cut-off in combination to a primer optimization step by multiplexing RT-LAMP. The study aimed to detect Orf7a and Orf3a genes simultaneously. After the optimization process, the overall test specificity was 94%. It is important to note that none of these papers refers to intermediate samples in the same way as we did and clearly observed during screening.

These observations led us to consider that the color determination and interpretation represents an important property in colorimetric RT-LAMP assays due to point-of-care glimpse, where no automated method is proposed to determine the results. Operator naked eye result determination should be clear between positive (yellow) and negative (pink) results, while intermediate color will be confusing and may produce an incorrect report or biased interpretation.

The mismatch results in RT-LAMP brought some questions about their connection to the intermediate color pattern. It was difficult to ensure RNA quality to every clinical sample as a repetition process, because the nasopharyngeal or saliva effective collection depends on the collector’s ability, the patient's condition and to the RNA extraction process. Therefore, for colorimetric RT-LAMP as a point-of-care application a strict internal control must be established. Controls are very consolidated in scientific literature and diagnostic procedures. The usage of internal control in RT-qPCR validates not only template but also molecular biology reagents quality and amount. Samples that are undetected for the target and for the internal control are invalidated, preventing false-negative results. Some SARS-CoV-2 RT-LAMP studies use an internal control from RT-qPCR (Lau et al. 2020; Osterdahl et al. 2020; Zhang and Tanner 2020) however, the objective is not performing any sophisticated additional technique and validation for the point-of-care application. With that in mind, the present study showed for the first time the essential role of the internal control in RT-LAMP context, evaluating the performance of the primers and the clinical relevance of the method.

With all the demonstrated findings, we can assert that colorimetric RT-LAMP SARS-CoV-2 assays should be performed with target tests in combination to an internal control as a validation procedure. Both tests analyzed here returned poor performance and sensitivity when low RNA amount was used, with RT-qPCR Ct > 30. Meyerson et al. 2020 is one of the few authors found in literature that actually performed RNA internal control for colorimetric RT-LAMP reactions. In their article they evaluated SARS-CoV-2 detection in saliva by colorimetric RT-LAMP and RNAse P as internal control, which was positive in all 463 clinical samples tested, but they did not describe the performance in comparison to RT-qPCR. Multiplex RT-LAMP able to detect SARS-CoV-2 multiple genes plus the internal control was already suggested by Meyerson et al. 2020 and Zhang and Tanner 2020. Although these reports describe how easy the implementation of a human RNA control is, recent and relevant publications seem to ignore it.

The days of symptoms data is an important finding regarding colorimetric RT-LAMP assay, emphasizing that colorimetric RT-LAMP for SARS-CoV-2 as a point-of-care retains application period. With this data we demonstrated that SARS-CoV-2 detection by colorimetric RT-LAMP as a point-of-care test is strictly related with the days of symptoms reported by the patients, suggesting that until five days of symptoms the use of colorimetric RT-LAMP is indicated, while with more than seven days, the method has a reduced sensitivity. Moreover, the performance of colorimetric RT-LAMP and RT-qPCR is comparable regarding the days of symptoms, with very similar results. Thus, colorimetric RT-LAMP correlates with RT-qPCR performance, as patients with Ct < 30 presented number of days after symptoms onset very similar to those that showed a positive colorimetric RT-LAMP, while samples with Ct between 30 and 35 presented symptoms days similar to intermediate/negative colorimetric RT-LAMP.

## Conclusions

Here we demonstrated that colorimetric RT-LAMP SARS-CoV-2 detection is an efficient, practical and reliable assay but has its limitations. The colorimetric method of this approach represents a positive property due to its simplicity but shows limitations in color determination and interpretation. Our recommendation for a realistic application of colorimetric RT-LAMP as a point-of-care diagnostic tool, is to always repeat the intermediate samples or perform a distinct technique, such as RT-qPCR to confirm the results. Despite the absence of an established Ct cut-off, La Scola et al. 2020 states that patients with Ct > 33 are not contagious, which reinforces that colorimetric RT-LAMP results are effective to detect patients that are able to spread the SARS-CoV-2 virus. Second, a high diagnostic sensitivity is reached when viremia is also high, mainly in the first week of symptoms (Cevik et al. 2020) which restrains the test to a limited timeline application but with great performance as a point-of-care for SARS-CoV-2 acute infections. Finally, we demonstrated the need for an internal control in parallel with SARS-CoV-2 detection in colorimetric RT-LAMP to validate the assay and avoid false-negative results. In the context of a pandemic situation, the lack of internal control and consequent generation of false-negative results may lead to mistaken diagnosis, increased virus spread, inappropriate or useless treatments and ultimately to an increase in the number of deaths.

## Supplementary Information

**Additional file 1: Table S1.** Set of primes designed by Rabe and Cepko (2020) to detect SARS-CoV-2 by RT-LAMP.

**Additional file 2: Figure S1.** Detection of SARS-CoV-2 by RT-qPCR in clinical samples, showing cycle threshold for 216 positive samples by E-gene detection (black dots) and for RNAse P internal controls detection for all 466 samples (red squares). Horizontal lines in both clusters represent the mean cycle threshold.

**Additional file 3: Figure S2.** Correlation between the number of days after symptoms onset.

## Data Availability

The datasets supporting the conclusions of this article are included within the article and its additional files.

## Abbreviations

COVID-19: Coronavirus disease 2019
RT-LAMP: Reverse Transcription Loop-mediated Isothermal Amplification
RT-qPCR: Quantitative reverse transcription-polymerase chain reaction
SARS-CoV-2: Severe acute respiratory syndrome coronavirus 2
