# Supplementary material for: Essential properties and pitfalls of colorimetric Reverse Transcription Loop-mediated Isothermal Amplification as a point-of-care test for SARS-CoV-2 diagnosis
Source: Mol Med. 2021 Mar 26;27:30. doi: 10.1186/s10020-021-00289-0 (PMC7996115; doi:10.1186/s10020-021-00289-0)
Supplement: Supplementary file 1 — Additional file 1: Table S1. Set of primes designed by Rabe and Cepko (2020) to detect SARS-CoV-2 by RT-LAMP. [file 10020_2021_289_MOESM1_ESM.docx]

**Table S1.** Set of primers designed by Rabe and Cepko (2020) to detect SARS-CoV-2 by RT-LAMP.

| F3 | CGGTGGACAAATTGTCAC |
| --- | --- |
| B3 | CTTCTCTGGATTTAACACACTT |
| LF | TTACAAGCTTAAAGAATGTCTGAACACT |
| LB | TTGAATTTAGGTGAAACATTTGTCACG |
| FIP | TCAGCACACAAAGCCAAAAATTTATTTTTCTGTGCAAAGGAAATTAAGGAG |
| BIP | TATTGGTGGAGCTAAACTTAAAGCCTTTTCTGTACAATCCCTTTGAGTG |
