# Supplementary material for: Essential properties and pitfalls of colorimetric Reverse Transcription Loop-mediated Isothermal Amplification as a point-of-care test for SARS-CoV-2 diagnosis
Source: Mol Med. 2021 Mar 26;27:30. doi: 10.1186/s10020-021-00289-0 (PMC7996115; doi:10.1186/s10020-021-00289-0)
Supplement: Supplementary file 2 — Additional file 2: Figure S1. Detection of SARS-CoV-2 by RT-qPCR in clinical samples, showing cycle threshold for 216 positive samples by E-gene detection (black dots) and for RNAse P internal controls detection for all 466 samples (red squares). Horizontal lines in both clusters represent the mean cycle threshold. [file 10020_2021_289_MOESM2_ESM.pdf]

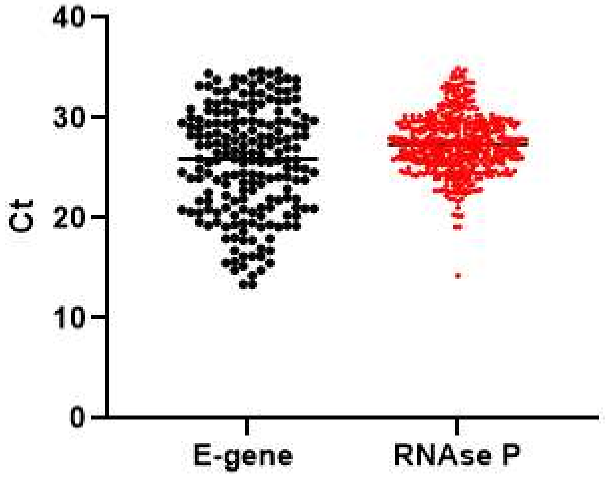

Figure S1. Detection of SARS-CoV-2 by RT-qPCR in clinical samples, showing cycle threshold for 216 positive samples by E-gene detection (black dots) and for RNAse P internal controls detection for all 466 samples (red squares). Horizontal lines in both clusters represent the mean cycle threshold.
