# Supplementary figures and images for: Essential properties and pitfalls of colorimetric Reverse Transcription Loop-mediated Isothermal Amplification as a point-of-care test for SARS-CoV-2 diagnosis
Source: Mol Med. 2021 Mar 26;27:30. doi: 10.1186/s10020-021-00289-0 (PMC7996115; doi:10.1186/s10020-021-00289-0)

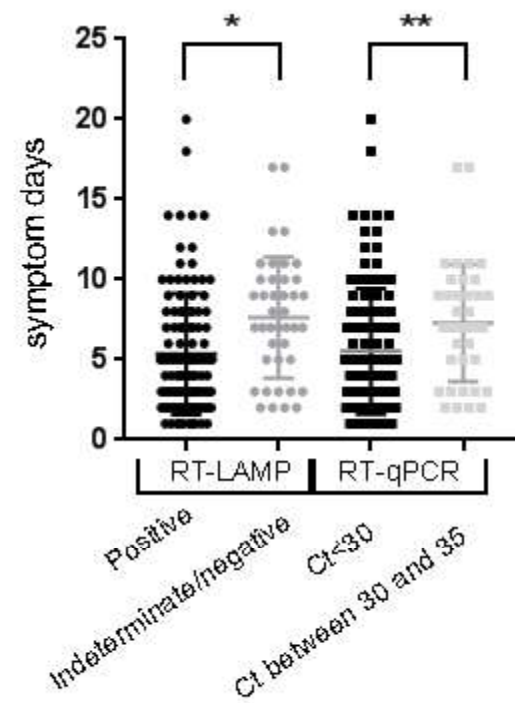

**Figure S2.** Correlation between the number of days after symptoms onset.

Supplement: Supplementary file 3 — Additional file 3: Figure S2. Correlation between the number of days after symptoms onset. [file 10020_2021_289_MOESM3_ESM.pdf]
